# A VLP Vaccine Induces Broad-Spectrum Cross-Protective Antibody Immunity against H5N1 and H1N1 Subtypes of Influenza A Virus

**DOI:** 10.1371/journal.pone.0042363

**Published:** 2012-08-07

**Authors:** Chia-Ying Wu, Yi-Chun Yeh, Jia-Tsrong Chan, Yu-Chih Yang, Ji-Rong Yang, Ming-Tsan Liu, Ho-Sheng Wu, Pei-Wen Hsiao

**Affiliations:** 1 Agricultural Biotechnology Research Center, Academia Sinica, Taipei, Taiwan; 2 Genomics Research Center, Academia Sinica, Taipei, Taiwan; 3 Institute of Biotechnology, College of Bio-Resources and Agriculture, National Taiwan University, Taipei, Taiwan; 4 Centers for Disease Control, Department of Health, Taipei, Taiwan; Public Health Agency of Canada, Canada

## Abstract

The recent threats of influenza epidemics and pandemics have prioritized the development of a universal vaccine that offers protection against a wider variety of influenza infections. Here, we demonstrate a genetically modified virus-like particle (VLP) vaccine, referred to as H5M2eN1-VLP, that increased the antigenic content of NA and induced rapid recall of antibody against HA_2_ after viral infection. As a result, H5M2eN1-VLP vaccination elicited a broad humoral immune response against multiple viral proteins and caused significant protection against homologous RG-14 (H5N1) and heterologous A/California/07/2009 H1N1 (CA/07) and A/PR/8/34 H1N1 (PR8) viral lethal challenges. Moreover, the N1-VLP (lacking HA) induced production of a strong NA antibody that also conferred significant cross protection against H5N1 and heterologous CA/07 but not PR8, suggesting the protection against N1-serotyped viruses can be extended from avian-origin to CA/07 strain isolated in humans, but not to evolutionally distant strains of human-derived. By comparative vaccine study of an HA-based VLP (H5N1-VLP) and NA-based VLPs, we found that H5N1-VLP vaccination induced specific and strong protective antibodies against the HA_1_ subunit of H5, thus restricting the breadth of cross-protection. In summary, we present a feasible example of direction of VLP vaccine immunity toward NA and HA_2_, which resulted in cross protection against both seasonal and pandemic influenza strains, that could form the basis for future design of a better universal vaccine.

## Introduction

Influenza infection is a vaccine-preventable disease. The neutralizing antibodies elicited by vaccination inhibit the enzyme activity of hemagglutinin (HA) and neuraminidase (NA) of influenza viruses, thus reducing virus replication and protecting against disease. The HA is the most abundant of the three integral membrane proteins (HA, NA, and matrix protein 2, M2) in the viral envelope and is responsible for both binding and fusion. At low pH within the endosome, HA undergoes great conformational changes that lead to fusion between the viral envelope and endosomal membrane, thus allowing the nucleocapsid to release into the cytoplasm to initiate viral replication. However, there is a perquisite that HA precursor (HA_0_) must be cleaved by host proteases to be active form before membrane fusion. After assembly into the trimeric form in the endoplasmic reticulum, each HA_0_ molecule is proteolytically cleaved into two subunits, HA_1_ and HA_2_, which remain linkage by a disulfide bond [1]. HA_1_ is responsible for binding to host cell receptors, whereas HA_2_ contains a stretch of hydrophobic amino acids which is known as the ‘fusion peptide’ and responsible for membrane fusion [2]. Therefore, the vaccine-raised antibodies against the HA_0_, HA_1_ and/or HA_2_ portions have known to inhibit or ameliorate the virulence of influenza infection. Usually, the HA antibodies elicited by natural infection with the seasonal influenza virus or vaccination predominantly target the receptor binding of the HA_1_ domain and consequently confer a sterilizing immunity to inhibit repeated infection by the cognate virus [3]. However, the emergence of new influenza viruses is inevitably associated with antigenic drift and/or shifts that result in epidemics or even pandemics. As a result, the specific neutralizing HA_1_ antibody corresponding to the previous circulating strains tend to lose their protective abilities. Therefore, development of a universal influenza vaccine is an important issue to be addressed and has been a priority of vaccine research [4].

The NA of influenza virus is pivotal in release and spread of progeny virions, following the intracellular viral replication process [5]. Given that the NA antigen has only a moderate rate of antigenic variation compared with HA, NA shows greater potential to be used as a target for a universal vaccine. The potential of NA in cross-protective immunity against influenza viruses has been widely recognized for several years and was recently discussed at a World Health Organization (WHO) meeting [6], [7], [8]. A DNA vaccine induced anti-huN1 immunity in animal models and conferred partial cross protection against the avian H5N1 virus [9]. Additionally, the observation of human sera cross-reactive with the NA of the H5N1 virus has raised the possibility that exposure to seasonal H1-serotyped influenza viruses or vaccines in the human population might elicit some degree of resistance to H5N1 infection [9]. Even though both HA and NA are essential elements of currently licensed vaccine preparations, the resulting immunity toward NA is rarely measured and is masked by antigenic competition with the stronger immunogenic response to HA [10], [11], [12]. Therefore, the durable effect of the humoral immunity induced by the NA antigen is difficult to quantify. In this study, we applied recombinant virus-like particle (VLP) technology to create an avian N1-VLP vaccine antigen that mimics the native structure of the NA spikes projecting from the parental virion. Through a comparative analysis of the humoral immunity induced by vaccination with NA-based VLPs and the HA-based VLP, we clearly illustrate the role of NA in virus cross protections.

Towards the ultimate goal of a universal vaccine and given the highly immunogenic and fully recombinant character of VLPs, we fused the conserved ectodomain of the viral M2 protein (M2e, 23 a.a. residues) at the N-terminus of HA in the H5N1-VLP. We generated an unanticipated NA-based VLP, referred to as H5M2eN1-VLP. Our studies of the H5M2eN1-VLP showed it had a cross protective effect to prevent death on mice from RG-14 (H5N1), A/California/07/2009 H1N1 (CA/07), and A/PR/8/34 H1N1 (PR8) infection. Comprehensive study of the immune response generated by H5M2eN1-VLP vaccination revealed three layers of immune protective effects: (i) the production of neutralizing HA and NA antibodies against the cognate strain, although the HAI titer fell below the seroprotection level; (ii) a marked increase in the neutralizing avN1 antibody and cross-protection against the viral strains which are phylogenetically closely related to the NA of H5N1 such as CA/07; (iii) to recall the viral-specific anti-HA_2_ antibody against heterologous stains during early infection. Taken together, we demonstrate the potency of a novel VLP vaccine on cross protection against both seasonal and pandemic influenza strains and present a paradigm of universal vaccine against influenza diseases.

## Results

### H5N1-VLP Confers Partial Cross Protection Against Antigenically Different Influenza A Viruses

**Figure 1:**
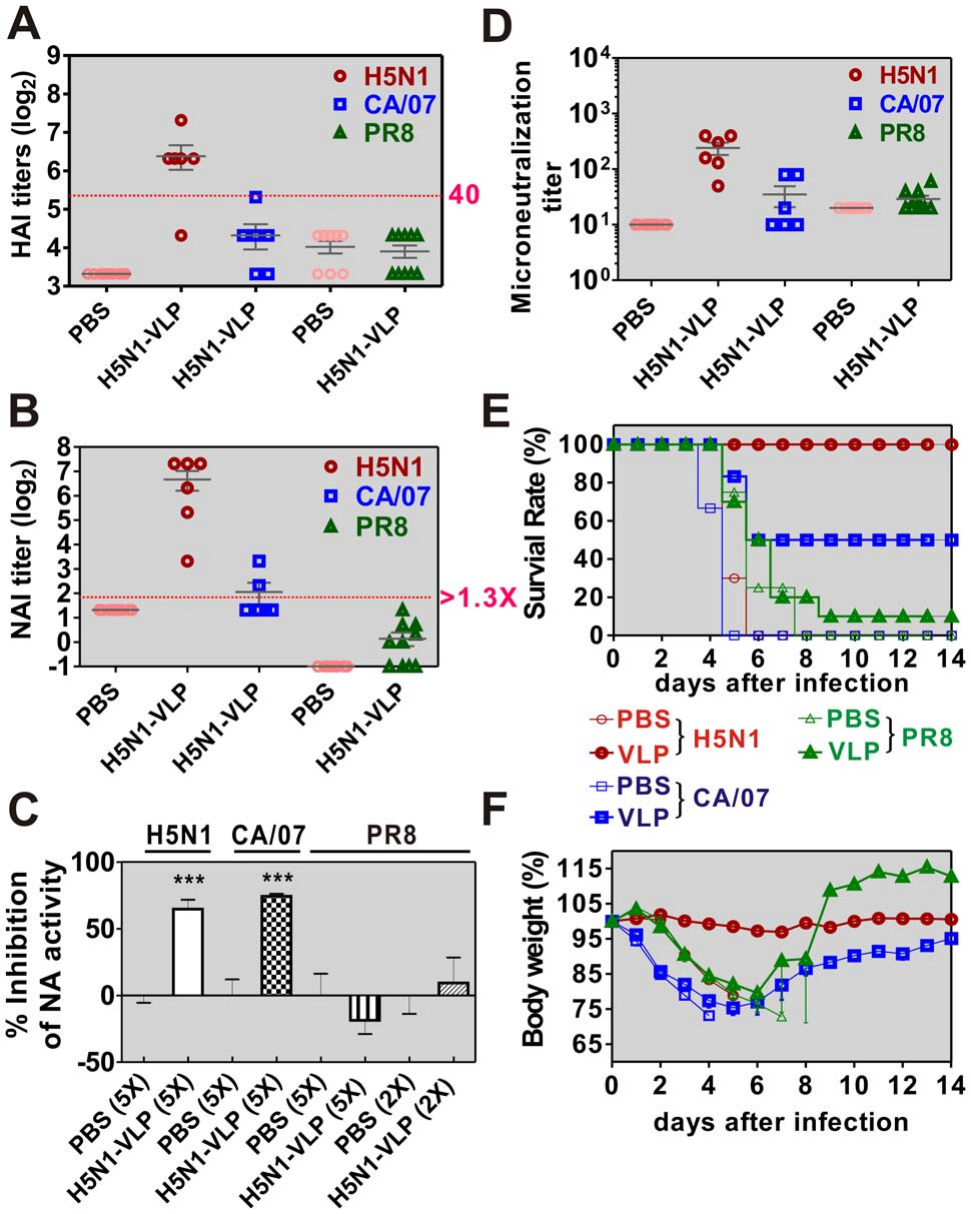
H5N1-VLP conferred protection against homologous and heterologous influenza viruses. Serum samples were collected at 2 weeks after a boosting vaccination. (A) The hemagglutination inhibition (HAI) titers of each H5N1-VLP vaccinated mouse against 4 HA units of H5N1, CA/07, and PR8 viruses, respectively. Data are presented as scatter plot with mean values ± SEM of the same group. HAI titer of 40 is set as threshold of seroprotection. (B) The miniaturized neuraminidase-inhibition (NAI) assays that measure the titers of neutralizing antibody against the NA of different viruses. NAI titer >1.3 fold was set as significant. (C) Reactivity of mice sera (five-fold or two-fold dilution) with the NA of H5N1 and H1N1 influenza viruses. The percentage of NA activity inhibition in each group is represented. Columns, means; bars, SEM. Comparing the vaccination and PBS control groups, asterisk (***), (**), and (*) indicates a significant difference (p<0.001), (p<0.01), and (p<0.05), by one-way ANOVA/Tukey’s range test. (D) Microneutralization assay to examine the neutralizing antibody response in vaccinated mice against H5N1, CA/07, and PR8 viruses. On day 42, vaccinated mice were challenged intranasally with a lethal dose (10× MLD_50_) of homologous or heterologous viruses as marked. (E) Survival rate and (F) body weight were monitored for 14 days post challenge.

Previously, we have demonstrated that the H5N1 virus-like particle (H5N1-VLP) is a potent and immunogenic vaccine antigen that confers full protection against the homologous influenza virus (RG-14) [13]. To evaluate whether the avian N1 (avN1) antibody can be elicited by H5N1-VLP and contribute to immune protection against N1-serotyped influenza viruses, groups of mice were immunized twice with 10 µg H5N1-VLP and then challenged with homologous RG-14 (H5N1) or heterologous CA/07 and PR8 viruses. The conventional hemagglutination-inhibition (HAI) assay was first applied to assess the functional titers of HA antibodies against different lineages of influenza virus. As expected, the HAI titers indicated seroprotection against the homologous virus, while the cross reaction with other H1 strains was not seroprotective (Figure 1A). Of the mice that received 10 µg H5N1-VLP vaccine, 83.3% produced HAI titers for the H5N1 virus over 1∶40, with the mean HAI titer reaching about 1∶80 (red circles). In parallel experiments, the reciprocal seroprotection rate for heterologous CA/07 and PR8 viruses dropped to 16.67% and 0%, respectively.

We next performed an extensive analysis of NAI titers induced by H5N1-VLP using the miniaturized NA inhibition assay [14]. NA-neutralizing antibody responses to the homologous avN1 antigen were detected in all mice, although the NAI titers may have been overestimated due to the HA-matched virus used in this assay. In the absence of the matched HA antibody, the cross-protective NAI titers elicited by H5N1-VLP to the heterologous swine N1 (swN1) and human N1 (huN1) were detected in 2 of 6 and 0 of 10 individuals, respectively (Figure 1B). A more sensitive detection method (NA-Star®Influenza Neuraminidase Inhibitor Resistance Detection kit) also verified the cross-reactivity of NA antibodies in the H5N1-VLP vaccinated mice, which was significant in the case of CA/07 but very weak in the case of PR8 (Figure 1C).

We next assessed the viral neutralizing activities mediated by antibodies through plaque reduction assays. Vaccination with H5N1-VLP induced high titers (mean = 240) of antibodies that neutralized the homologous H5N1 virus, whereas the effects on the heterosubtypic CA/07 and PR8 viruses were limited, with respective mean titers of 35 and 29 compared to the background (PBS) group with titers of 10 and 20 (Figure 1D). Corresponding to the vaccine effects reflected in the NAI and microneutralization assays, 100%, 50%, and 10% of vaccinated mice survived the 10× MLD_50_ challenge with H5N1, CA/07, and PR8 viruses, respectively (Figure 1E). Consistent with our previous report, all mice challenged with homologous virus survived with no obvious loss in body weight (Figure 1F, red circles) [13], while the heterosubtypic CA/07 challenged mice lost as much as 25% body weight, but began to recover 7 days post-infection (Figure 1F, blue block). This raised the question whether the anti-avN1 immunity induced by the VLP vaccine is capable of broadening the protective effects against N1-serotyped influenza viruses.

### Construction and Characterization of NA-based VLPs

To address the cross-protective character of VLP-induced anti-N1 immunity and enhance the production of cross-reactive antibodies against the M2e conserved epitope, we constructed an avN1-VLP and a recombinant modified H5M2eN1-VLP. The former was generated by stable co-expression of the N1, M1, and M2 genes in Vero cells. The H5M2eN1-VLP was created through genetic modification by inserting the M2e sequence into the N-terminus of HA, which is designed to boost anti-M2e antibody production after vaccination.

**Figure 2:**
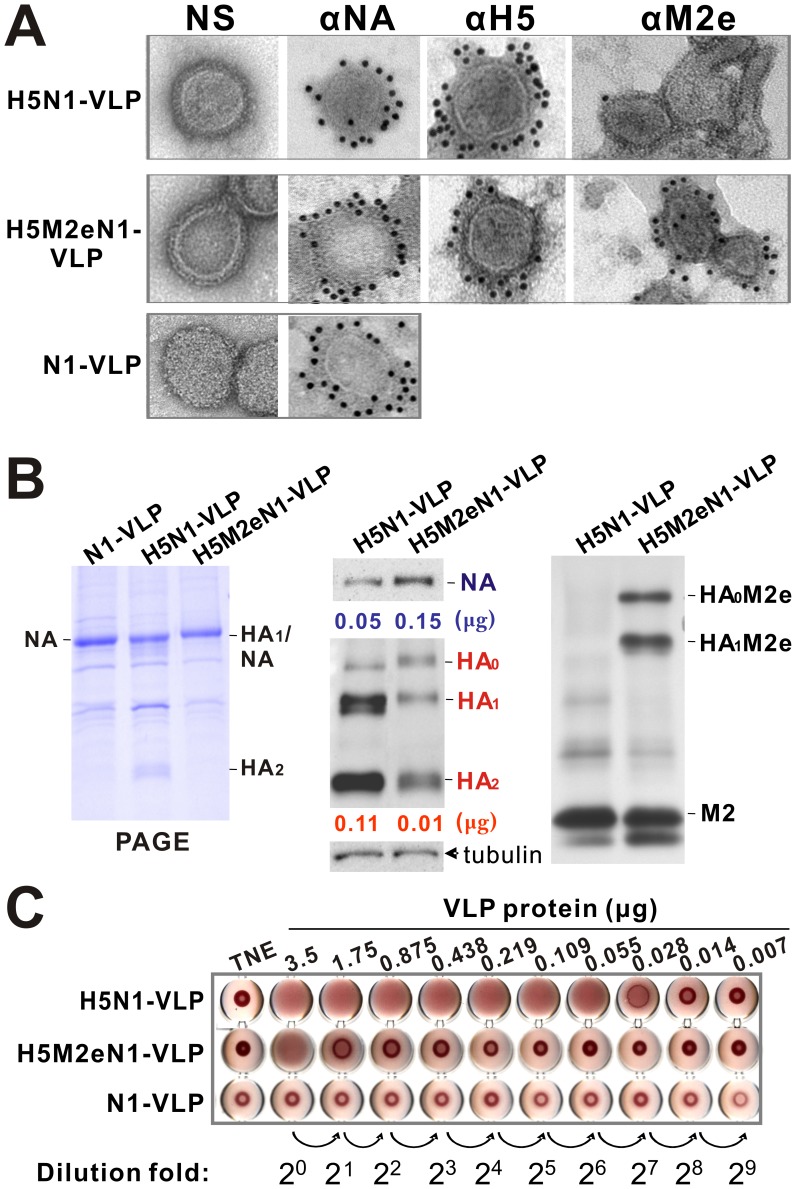
Comparative characterizations of N1-based VLPs with their prototyped H5N1-VLP. (A) Secreted VLPs of different designs are as indicated to the left of panels. Purified VLPs were adsorbed onto formvar/carbon-coated nickel grids. NS, negatively stained with 2% uranyl acetate; immunogold stained with specific antibodies as marked on the top. The secondary antibodies were goat anti-rabbit or anti-mouse conjugated to 12 nm gold beads. The grids were observed by TEM at 100,000× magnification. (B) Western blot analysis of virus-specific proteins in VLPs. Equal amount of VLPs were separated on a 7.5–12.5% gradient gel followed by Coomassie blue staining or western blot analysis with specific antibodies. Identity of viral proteins in the VLPs were detected and labeled on the right. The same blot was probed with anti-tubulin antibody as a loading control of VLP preparations. *Middle*, the HA and NA amount in 0.5 µg of each VLP was marked below. (C) Assessment of HA function by hemagglutination assay. The amounts of VLPs used are indicated, in a two-fold serial dilution. TNE (buffer of VLPs) was used as the negative control. The antibodies used in this study were tubulin (ab6160), N1 (ab21305), and M2e (ab5416) from Abcam (Cambridge, MA). Rabbit polyclonal antibody against H5 was provided by Dr. Che Ma (Genomics Research Center, Academia Sinica).

To investigate whether the N1-VLP and H5M2eN1-VLP assemble and bud from the individual VLP-producer cell lines, conditioned media was collected and purified by co-sedimentation in sucrose density gradient centrifugation. The morphology and antigen presentation of modified VLPs were compared to the prototypical H5N1-VLP using transmission electron microscopy (TEM) after staining with 2% uranyl acetate or immunogold (Figure 2A). The modified H5M2eN1-VLP displayed a generally spherical morphology similar to H5N1-VLP and N1-VLP (Figure 2A, NS). The release of N1-VLP from VLP-producer cells is in accordance with the results obtained by Lai and colleagues [15]. The surface HA and NA glycoproteins on H5N1-VLP and H5M2eN1-VLP were immunogold labeled in parallel with specific antibodies as marked (Figure 2A). We found that the surface of the H5M2eN1-VLP had increased levels of N1 and M2e epitope but less HA than the H5N1-VLP (Figure 2A, αNA, αM2e, αH5). We quantified the HA and NA proteins in H5N1-VLP, H5M2eN1-VLP, and N1-VLP by western blot analyses (Figure S1A, B) and their relative abundances to total VLP protein are summarized in Table 1. Compared to the H5N1-VLP, the HA content of the H5M2eN1-VLP decreased by 10 fold while the NA content tripled (Figure 2B, left and middle). Additionally, immuno-detection of the H5M2eN1-VLP with M2e antibody by western blotting and immuno-EM showed the M2e epitope indeed fused with the HA_0_ and HA_1_ molecules and was displayed on the spikes of the H5M2eN1-VLP (Figure 2B, right and Figure 2A, αM2e). We also examined the HA activities in the three types of VLPs by HA assay and found that the HA unit was decreased from 2^6^ fold in the H5N1-VLP to 2^0^ fold in H5M2eN1-VLP (Figure 2C). These results demonstrate that H5M2eN1-VLP and N1-VLP are both N1-dominant VLPs. This shift in the antigenic content of the VLP from predominantly HA to predominantly NA could potentially shift the immune response from HA towards NA, which might help define the cross-protection conferred by NA in the VLP-induced immunity.

**Table 1:** The relative abundance of HA and NA attributed to total VLP proteins.

VLP vaccine	H5N1-VLP	H5M2eN1-VLP	N1-VLP
HA (%)	22.5±5.97	2.2±0.19	0
NA (%)	10.9±0.28	29.5±8.58	38.6±10.91

### Humoral Immune Response of N1-based VLPs

**Figure 3:**
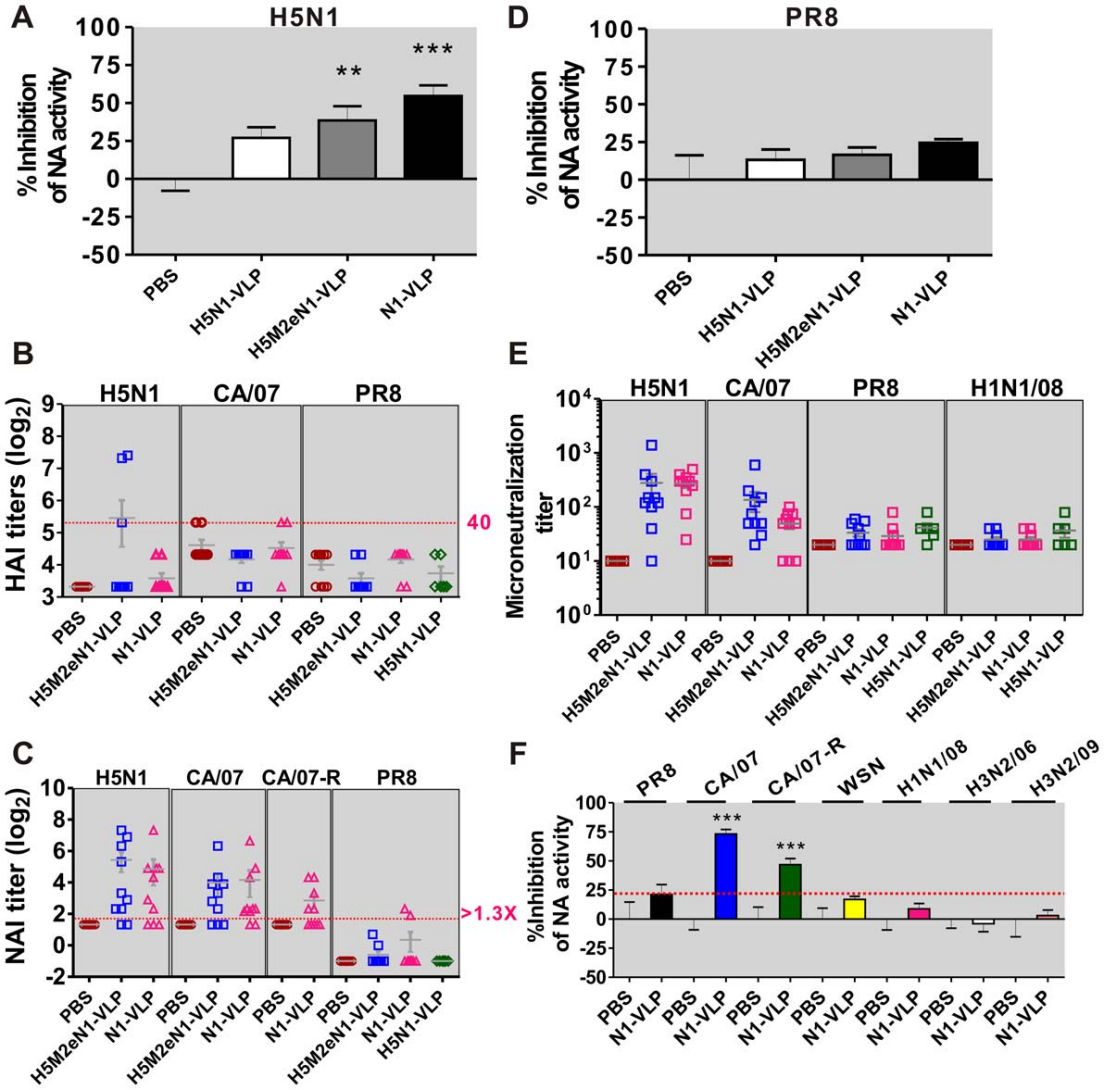
N1-based VLPs induced cross-reactive humoral immune responses. (A) VLP-induced avN1 antibodies against the NA activity of the homologous H5N1 virus. Sera were collected from mice immunized with 15 µg dosages and types of VLPs as labeled and diluted tenfold for NA inhibition assay. (B) HAI titers and (C) NAI titers induced by N1-based VLPs against homologous and heterologous viruses were determined. The viruses are indicated above. (D) Antisera elicited by different VLPs were fivefold diluted and examined for the inhibition of NA activity derived from PR8 virus. (E) Microneutralization assay to assess the humoral immune responses induced by the VLP vaccines against viruses as labeled. (F) Effect of antisera (tenfold dilution) from N1-VLP vaccinated mice on the NA activity of heterologous strains of influenza virus. The NA antigen derived from N1- or N2-serotyped influenza viruses are labeled at top. CA/07-R is a clinical virus strain with a Tamiflu-resistant phenotype. A/Taiwan/9042/2008(H1N1) (H1N1/08), A/Taiwan/83/2006(H3N2) (H3N2/06), and A/Taiwan/4055/2009(H3N2) (H3N2/09) are clinical virus strains isolated from patients in Taiwan. Comparing the vaccination and PBS control groups, asterisk indicates statistic significance as used in Figure 1.

To determine whether avN1-based VLPs can boost the anti-N1 immunity against N1-serotyped viruses, we immunized mice twice with 15 µg of H5N1-VLP, H5M2eN1-VLP and N1-VLP and evaluated the humoral immune responses. As compared to H5N1-VLP, vaccination with either one of the N1-based VLPs (H5M2eN1-VLP or N1-VLP) induced greater NA inhibition activity against the cognate NA (Figure 3A). Further, immunity against different strains of influenza A viruses conferred by the N1-based VLP vaccines was analyzed by measuring HAI, NAI, and microneutralization titers. As predicted, the two N1-based VLPs did not produce sufficiently high titers of HA protective antibodies against homologous H5N1, heterologous CA/07, or PR8 viruses (Figure 3B). However, substantial NAI titers against H5N1 and CA/07 were elicited by the two N1-based VLP vaccines, but reactivity with the heterologous N1 of the PR8 strain was detected in less than 20% (N = 10) of the vaccinated mice (Figure 3C). The NAI titer data and results of the NA inhibition assays against PR8 were consistent and there was no statistical difference between groups of vaccinated and PBS control mice (Figure 3C, D).

The microneutralization results revealed that the H5M2eN1-VLP and N1-VLP vaccinations stimulated a highly potent neutralization response against the H5N1 virus (mean titers of 279 and 270, respectively) and CA/07 virus (mean titers of 135 and 49, respectively), in which the PBS group represented the background titer (mean  = 10) (Figure 3E, H5N1 and CA/07). For the PR8 virus, the mean titers of neutralizing antibodies elicited by individual vaccinations with 15 µg H5M2eN1-VLP, N1-VLP, H5N1-VLP, or mock-vaccinated (PBS) were about 34, 29, 42, and 20, respectively (Figure 3E). Also, no significant viral-neutralization against other human-derived H1N1 virus was detected in the vaccinated mice compared to the PBS control group (Figure 3E, H1N1/08, A/Taiwan/9042/2008/H1N1: a circulating seasonal influenza A/H1N1 virus in Taiwan). These results suggest that N1-based VLPs predominantly elicit an NA humoral immune response to neutralize the H5N1 and CA/07 viruses, but the antibodies moderately impeded the infection of human-derived H1N1 viruses. To more extensively determine whether the anti-avN1 antibodies elicited by N1-VLP cross-react with the NA from other subtypes, the H1N1- and H3N2-serotyped viruses, in addition to a clinical isolate from 2010 with Tamiflu-resistance (CA/07-R), were used as targets for NA inhibition assays. As shown, only the NA activities derived from CA/07 and its cognate Tamiflu-resistant strain, CA/07-R, could be obviously impeded by the avN1 antibody (Figure 3F). Based on these data, we suggest that N1-based VLP vaccines might confer protections against H5N1 and CA/07 challenge as well as against the drug-resistant CA/07-R H1N1 virus depending on the NA humoral immunity.

### H5M2eN1-VLP Vaccine Broadens the Spectrum of Protection

**Figure 4:**
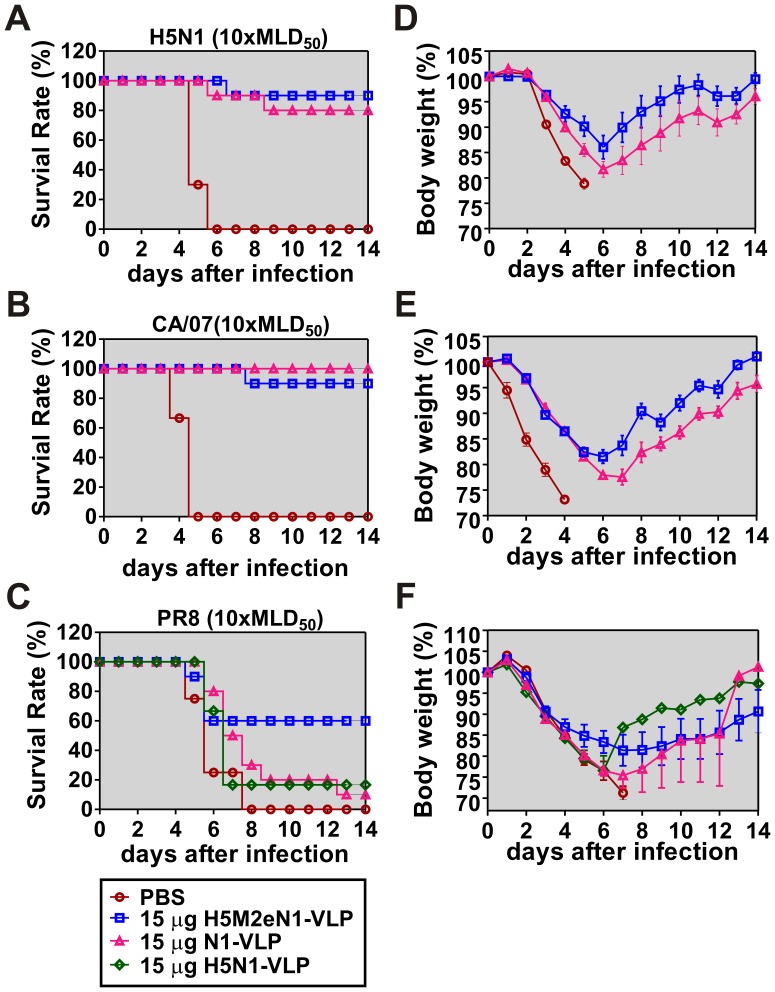
H5M2eN1-VLP vaccination broadens heterologous protections. Mice groups (n = 6–10) immunized with various VLP vaccines and infected with a lethal dose (10× MLD_50_) of reassortant RG-14 (H5N1) (A), CA/07 (B), or PR8 (C) influenza viruses. Body weight changes after infection with H5N1 (D), CA/07 (E), or PR8 (F) were recorded daily and plotted independently. The used dosage and individual VLP vaccines were as indicated.

To examine the cross-protective efficacy of the VLP vaccines *in vivo*, following prime-and-boost immunizations with H5M2eN1-VLP or N1-VLP, mice were challenged with a lethal infection (10× MLD_50_) of H5N1, heterosubtypic CA/07 or PR8. All mice in the mock-vaccinated group died from viral infections within 4 to 7 days depending on the viral strains (Figure 4A, B, and C; PBS). After viral infection, H5M2eN1-VLP vaccination resulted in survival rates of 90% for H5N1, 90% for CA/07, and 60% for PR8 (Figure 4A, B, and C; H5M2eN1-VLP). Upon infection with homologous H5N1 virus, the mice receiving the H5M2eN1-VLP have initially lost weight and subsequently recovered, suggesting an immune response which “caught up” with an established infection involved to alleviate the symptoms and accelerate the recovery rather than directly sterilize the virus. And, the major reason which makes the H5M2eN1-VLP decreasing the ability of viral clearance compared with H5N1-VLP could be due to ten-fold reduction of HA content in modified VLPs (Figure 2B, middle). The N1-VLP vaccine protected 80% of mice fatality against H5N1 and 100% against CA/07, but only 10% of mice infected with PR8 (Figure 4A, B, and C; N1-VLP). In this case, the avN1-VLP eliciting significant cross-reactive NAI titers against the NA of CA/07 virus provided 100% protection from death, proposed that anti-avN1 immunity played a critical role in mice recovery during CA/07 viral infection. Additionally, to compare the cross-protective efficacy of similar dosages of VLP vaccines against PR8, two doses of 15 µg H5N1-VLP were administered to mice in parallel. Of this vaccination group, 16% survived the PR8 challenge, which was similar to that observed with vaccination with 10 µg H5N1-VLP (Figure 4C vs. 1E). Ultimately, the cross-protection conferred against PR8 by the H5M2eN1-VLP vaccine, with a survival rate of 60%, was superior to that provided by the H5N1-VLP and N1-VLP vaccines, with survival rates of 16% and 10%, respectively. However, the wide range of cross-protection induced by H5M2eN1-VLP vaccination against PR8 cannot be attributed completely to the HAI or NAI effects of the antibodies. After lethal infections with individual viruses, the H5M2eN1-VLP-vaccinated mice regained body weight more rapidly, while all mice immunized with N1-VLP continued to lose more body weight (Figure 4D, E, and F).

These data suggest the possibilities that cell-mediated immune response and/or antibodies against the conserved region of viral proteins such as M2e or HA_2_ elicited by H5M2eN1-VLP vaccination may also be involved in accelerating the recovery from heterologous viral challenges.

### Th-1 and Th-2 Cytokine Responses in Mice Immunized with VLP Vaccines

**Figure 5:**
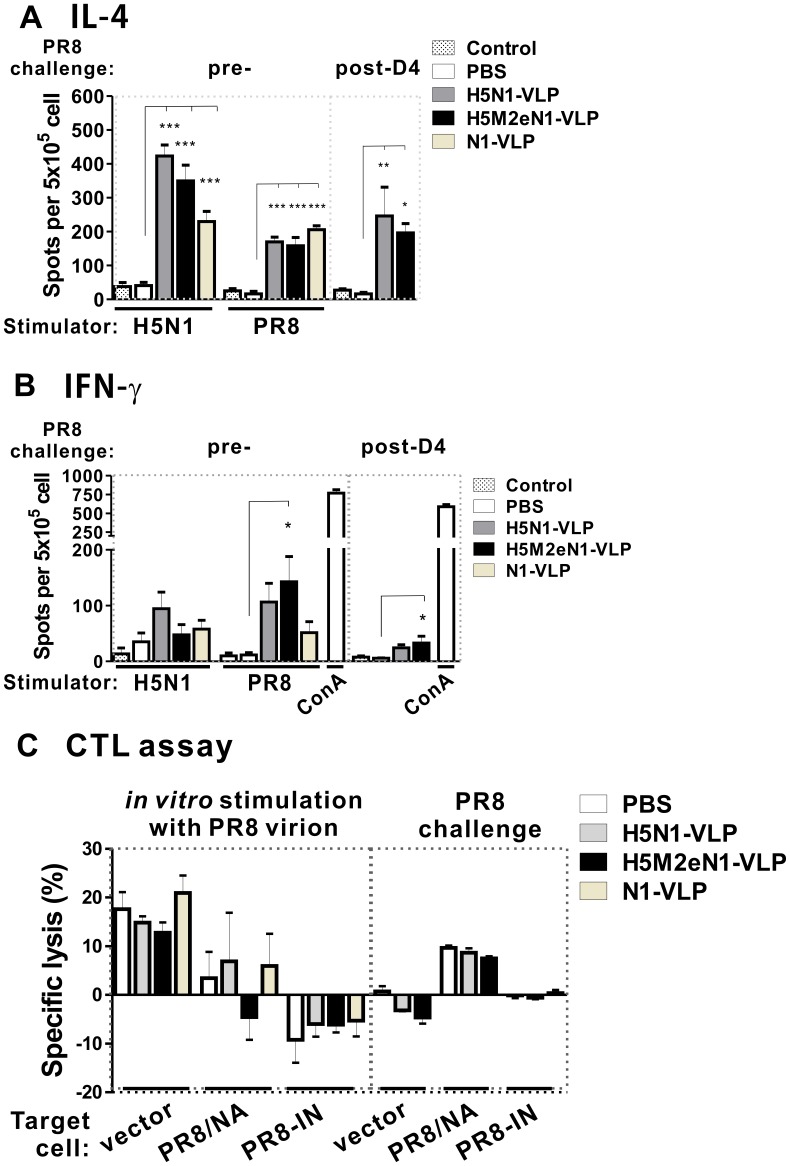
T helper cell responses in VLP-vaccinated mice. The level of IL-4 (A) and IFN-γ (B) spot-forming cells/5×10^5^ in spleens of mice was determined by ELISpot assay. Mice groups (n = 5) were immunized as described in the text either with 15 µg of VLPs vaccines or mock-treated (PBS). Splenocytes were stimulated *in vitro* with BEI-inactivated whole viruses of H5N1 (10 µg/mL), PR8 (4 µg/mL) or alternatively challenged with PR8 virus for four days (post-D4) as labeled on the top of A and B. The cells were stimulated with ConA (2.5 µg/mL, 1×10^5^ splenocytes/well) as a positive control for IFN-γ ELISpot assay or were mock stimulated as negative control (Control) for both ELISpot assays. Bars represent means ± SEM of spot counts in triplicate wells. Comparing the vaccination and PBS control groups, asterisk indicates statistic significance as used in Figure 1. (C) Cytotoxic T lymphocytes (CTL) responses to VLP vaccinations. Data shown were obtained at an effector/target ratio of 100∶1. Each bar represents the percentage of specific lysis (mean ± SEM). The used autologous target cells (4T1) infected with PR8 (PR8-IN) or either transduced with recombinant lentivirus expressing NA of PR8 (PR8/NA) or wild-type lentivirus (vector) were labeled. The splenocyte effectors prepared from the mice vaccinated with VLP vaccines or mock-treated (PBS) were *in vitro* stimulated with BEI-inactivated PR8 virion (4 µg/mL) for 5 days or *in vivo* challenged with PR8 virus for 4 days as indicated.

To investigate whether the cell-mediated immunity operate as a correlate of protection against disease, the type of cellular immune response induced by the VLP vaccines were characterized. After boosting immunization or 4 days post-infection with PR8, homologous and heterologous T-helper cell responses were evaluated by mouse IL-4 (Th-2) and IFN-γ (Th-1) ELISpot analyses. Spleen cells were collected from mice groups 7 days after the secondary bleeding (day 42) and *in vitro* stimulated with homologous (H5N1) or heterologous (PR8) influenza virions or 4 days after PR8 challenge. Both homologous H5N1 and heterologous PR8 strains stimulated robust response of IL-4 secretion *in vitro* (Figure 5A). *In vivo*, infection with PR8 virus stimulated IL-4 secreting cells in the spleen of mice vaccinated with both H5N1-VLP and H5M2eN1-VLP (Figure 5A, post-D4). In comparison with IL-4 response, the *in vitro* and *in vivo* IFN-γ responses were much less profound. Numbers of IFN-γ secreting cells specific for the viral antigens were lower than that of IL-4 secreting cells across all vaccine groups (Figure 5A, B). Significant Th-1 type responses were observed only in the H5M2eN1-VLP immunized mice following stimulation with heterologous PR8 virus *in vitro* or *in vivo* (Figure 5B, PR8 and post-D4). Th-1 type response in H5N1-VLP vaccine group was detectable but not statically different from the mock vaccine (PBS) group regardless of stimulation with homologous or heterologous viruses (Figure 5B, H5N1, PR8, and post-D4).

The virus-specific cytotoxic T lymphocyte (CTL) activity has been studied extensively to be important in much broader control of influenza infection via direct cytolysis of virus-infected cells. To further address whether CTL response is involved with the cross-strain protection of VLP vaccines, the splenocytes of mock-treated and VLP vaccinated mice were colleted following *ex vivo* stimulated with PR8 virion or PR8 post-challenge to evaluate the cytolytic activity against target cells that were either infected with PR8 virus or expressing the NA protein of PR8. As shown in Figure 5C, no differences in T cell cytotoxicity were detected among mice receiving with all VLP vaccines or mock-treated (PBS) for PR8-infected mouse 4T1 cell (PR8-IN) as well as 4T1 target cells expressing PR8 NA (PR8/NA) or empty cassette (vector). Overall, the limited IFN-γ secreting cells and undetectable CTL responses from splenocytes isolated from the mice received any of the three VLP vaccines suggest a predominant Th-2 response recalled by VLP vaccination following infection with homologous and heterologous viruses.

### H5M2eN1-VLP Vaccination Induced Rapid Recall Anti-HA2 Response to Influenza Infection

To determine whether anti-M2e or anti-HA_2_ antibodies contribute to the cross-protection of H5M2eN1-VLP vaccine against the PR8 virus, we examined the immune responses to M2e or HA_2_ before and four days after lethal infection (10× MLD_50_). To evaluate the anti-M2e response, mice sera was examined by ELISA against synthetic M2e peptide (2 µg/mL) coating in a 96-well plate. Compared to a commercial anti-M2e antibody used as a positive control, no difference in response against an M2e peptide was detected in mice sera regardless of H5M2eN1-VLP or mock vaccination before or after viral infection (data not shown). These results ruled out the involvement of an anti-M2e antibody response following H5M2eN1-VLP vaccination and viral infection.

**Figure 6:**
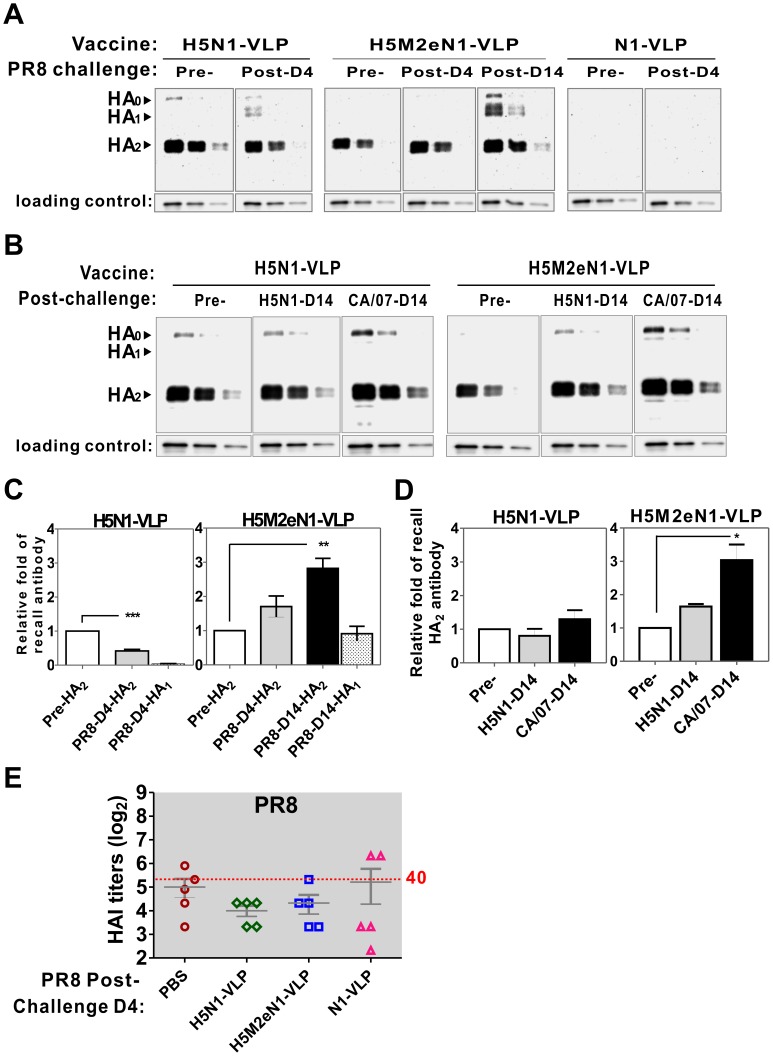
Recall antibody response induced by VLP vaccination to influenza. (A) Western blot analysis of VLP-induced humoral immunity against PR8 HA. The PR8 HA_0_ (11684-V08H, Sino Biological Inc.; expressed by baculovirus system) was treated with TPCK-trypsin (5 µg/mL) at 37°C for 15 min to induce cleavage into HA_1_ and HA_2_. The cleaved PR8 HA was admixed with purified H3N2-VLP (providing annexin A2 as loading control), serially diluted in two folds and quantified by western blot analysis. The antibody against the annexin A2 (ab41803, Abcam) in H3N2-VLP was used to normalize the loading amount of protein samples and transfer efficiency of western blotting. Mice vaccinated with various VLPs were bled before (Pre-), 4 days (Post-D4) or 14 days (Post-D14) after challenge as indicated. The antisera (500-fold dilution used in this assay) were analyzed by western blotting. HA_0_, HA_1_, and HA_2_ are labeled on the left. (B) Comparative analysis of cross-reactive anti-HA_2_ antibody elicited by VLP vaccination before challenge or recalled after homologous and heterologous viral challenges. VLP vaccines and strains of challenge virus are as indicated. (C), (D) The relative folds of antibody response specific to PR8 HA_1_ or HA_2_ in panel A and B were quantified and summarized correspondingly. Comparing the relative level of HA_2_ antibody before and after viral challenge, asterisk indicates statistic significance as used in Figure 1. (E) The HAI titers in vaccinated mice 4 days after PR8 infection were determined by standard methods. Scatter plot with mean values of the same group; bars, SEM. Type of VLP vaccine or PBS control is indicated.

To determine whether anti-HA2 antibodies can be elicited by VLP vaccination or stimulated by viral infection, we performed a western blot analysis, probing the vaccinated mice sera collected before or after PR8 viral challenge with PR8-derived HA. To quantify the production of anti-HA_2_ antibody, PR8 HA_0_ was proteolytically cleaved with trypsin and admixed with the H3N2-subtyped VLP which provides integral host proteins such as annexin A2 as a loading control. Following serial twofold dilution, the protein sample was analyzed by western blotting with different mice antisera. The quantification of HA_1_ and HA_2_ bands was normalized against the respective loading control (annexin A2) of each lane as detailed in dataset exported to the files of Excel S1 and S2. Before viral infection, the sera of mice that received immunization with H5M2eN1-VLP showed reactive against HA_2_ of PR8, and PR8 infection induced an early recall antibody response specific to the HA_2_ of PR8 (Figure 6A, H5M2eN1-VLP). Mice vaccinated with H5N1-VLP also produced antibodies cross-reacted with the HA_2_ of PR8 before viral challenge, but the antibody declined at 4 days after PR8 infection in concomitant to induction of anti-HA_1_ of PR8, (Figure 6A, H5N1-VLP). However, the recalled anti-HA_1_ antibody specific to PR8 virus was not sufficient to achieve seroprotection in the HAI assay during early infection (Figure 6A and C, H5N1-VLP). As for the antibody profiles of mice that received N1-VLP or PBS, western blot analysis revealed no signal against PR8 HA antigen in either group of mice sera before or 4 days after challenge (Figure 6A, N1-VLP). These results suggest that H5N1-VLP vaccination lacked recall anti-HA_2_ response to influenza virus, thereby failing to protect against heterologous PR8 challenge. The outcome indicates that H5M2eN1-VLP vaccination-induced recall antibody specific to the HA_2_ of PR8 after cognate viral infection is similar to the result of previous study. The immunization with a vaccinia virus recombinants expressing HA_2_ of the same HA subtype as the challenge virus (and HA_1_ of a different subtype) protected mice against lethal infection with a low dose (0.7× MLD_50_) of challenge virus [16]. In our case, because cleavage of the M2e-HA fusion protein in the VLP was markedly compromised, such a fusion might partially shelter the cleavage site of HA_0_ and preserve the prefusion structure of HA_2_, thus promoting antibody production against highly conserved HA_2_ region following viral infection (Figure 2B, HA_0_).

To understand whether the stimulation of anti-HA_2_ after viral infection is a common phenomenon associated with vaccination of H5M2eN1-VLP in mice, the sera of mice vaccinated with H5M2eN1-VLP or H5N1-VLP were analyzed by western blot before and 14 days after challenge with homologous H5N1 and heterologous CA/07 viruses. As shown in Figure 6B (H5M2eN1-VLP), cross-reactive antibodies against PR8 HA_2_ recalled following either H5N1 or CA/07 viral challenge. However, H5N1-VLP vaccination caused decrease in production of cross-reactive anti-HA_2_ antibodies after homologous H5N1 challenge and shown no great improvement on inducing of anti-HA_2_ antibody after heterologous CA/07 viral infection (Figure 6B, H5N1-VLP). The relative folds of HA_1_ and HA_2_ antibodies before and recall by post-infection between VLP vaccine groups in Figure 6A, B were quantified and summarized in Figure 6C, D, respectively. In conclusion, the H5M2eN1-VLP vaccine is unique in that it quickly stimulated the production of viral specific anti-HA_2_ antibodies in response to infections at an early stage and further improved recovery from viral lethal challenge.

In this study, we demonstrated the potential of the novel H5M2eN1-VLP as a universal vaccine candidate due to its ability to induce robust humoral immunity against multiple viral proteins including HA_1_, slowly drifting N1, and the highly conserved HA_2_ along with producing moderate level of IFN-γ upon heterologous viral challenge, which was successful in protecting mice from death after homologous and heterologous viral lethal infections.

## Discussion

Currently licensed influenza vaccines that are based on the variable HA antigens and elicit HA_1_-dominant neutralizing antibody provide only limited protection against homologous virus strains and offer insufficient protection against future pandemic outbreaks. Guarding against these potential pandemics necessitates annual vaccine updates to protect against newly emergent influenza viruses. A recombinant VLP vaccine platform provides the advantages of rapid response and scalable production to fight emerging influenza viruses since it relies on neither egg availability nor the use of live virus for vaccine manufacturing. Herein, we demonstrate that mammalian-expressed VLPs elicited protective antibodies against HA and NA and cross-protected against homologous and some heterologous viral infections. Vaccination with N1-VLP or H5M2eN1-VLP can induce high titers of NA-neutralizing antibody and provide protective humoral immunity against homologous H5N1 and heterologous CA/07 viral diseases. We theorize that the anti-avN1 immunity that resulted in improved mice survival following infection with heterologous N1-serotyped influenza viruses such as pandemic CA/07 H1N1 might have resulted from two possibilities: the phylogenetic similarity of the NA gene in avian H5N1 and swine-origin CA/07 H1N1 or the highly conserved surface exposed region in the NA structure shared by the H5N1 and CA/07 viruses [17], [18]. Moreover, the NA activity of a Tamiflu-resistant cognate of the CA/07 strain was significantly inhibited by the anti-avN1 antibody, suggesting that the NA antibody might remain reactive to the mutations selected against by clinical NA inhibitors. Additionally, the importance of NA-induced immunity has been associated not only with amerlioration of the illness and pathological effects following infection, but also prevention of secondary bacterial infections [9], [19], [20]. It also has been reported that the NIBRG-14 vaccine conferred better protection to the vaccinated hosts when it elicited both anti-HA and anti-NA antibodies, suggesting the measurement of both HA and NA antibodies may be essential for the accurate evaluation of vaccine efficacy [21]. Therefore, our results are in accordance with previous studies and support the notion that NA is as critical an antigen as HA for vaccine-induced immunity and should be integrated into the requirement or criteria for influenza vaccines that could extend the protection beyond the single annual strain, alleviate the associated syndrome from viral infection, or prepare for pre-pandemic influenza viruses.

Beyond the humoral NA immunity elicited by NA-based VLP vaccination, the modified recombinant H5M2eN1-VLP also rapidly recalled the anti-HA_2_ antibody response to viral infection, which provided substantial cross-protection against a different lineage of seasonal influenza virus (PR8). Currently, there are several vaccine strategies being investigated in the development of a universal vaccine such as immunization with conserved proteins or peptides or injection of DNA plasmid-encoding conserved protein sequences of influenza viruses to elicit neutralizing antibodies against various influenza strains. Among these approaches, vaccine candidates that direct immunity against the highly conserved stalk region of HA_2_ have proven promising as universal vaccines [16], [22], [23], [24], [25]. In agreement with this, immunization with plasmid DNA encoding the HA of the H1N1 virus followed by boosting with a trivalent seasonal vaccine stimulated the production of broadly neutralizing antibodies and conferred protection against divergent H1N1 viruses in mice and ferrets; these effects have been attributed to antibodies blocking the conserved stalk region of HA_2_ [23]. In parallel, several groups have designed HA_2_-based immunogens or a genetically modified headless-HA pseudotyped VLP as targets, all of which can induce cross-protective immune response in mice and provide partial protection against heterologous virus strains [22], [24], [25]. Building from these concepts, we show here that the novel H5M2eN1-VLP vaccine elicited viral-specific anti-HA_2_ antibodies depending on viral infection and mediated a more effective recovery from lethal infection (10× MLD_50_) against a heterologous subtype of influenza virus. Since the variable immunogenic HA_1_ region was disrupted by treatment with dithiothreitol or genetic deletion, the immune dominance of HA_1_ was weakened, which increased the likelihood of eliciting an antibody against the highly conserved HA_2_ [25], [26]. The H5M2eN1-VLP vaccination induced the production of antibodies against the conserved epitopes of HA_2_ upon viral infection is likely due to the N-terminal fusion with M2e on HA of the VLP vaccine. As shown in X-ray crystallography structure study of the HA precursor (HA_0_), the cleavage site resides in an extended, highly exposed surface loop, near the viral membrane and beside the N-terminus of HA where the M2e was placed [27]. Hence, the M2e fusion in H5M2eN1-VLP may obscure the HA_0_ cleavage site, thus resulting in more HA_2_ presented for antibodies to be raised against during viral infection. Previous studies have shown that the HA_2_ antibodies do not prevent the attachment of virus to the cell surface, which supports the possibility of low microneutralization titers against human-derived H1N1 viruses (Figure 3E, PR8 and H1N1/08). However, the HA_2_ antibodies can also influence the second stage of infection, including blockage of membrane fusion, prevention of HA_1_/HA_2_ cleavage, or activation of infected cell and virus particle clearance by antibody-dependent cell mediated cytotoxicity mechanisms, which contribute to a milder course of infection and improved survival [16], [28], [29], [30]. Taken together, our finding indicates that stable structure of HA_0_ spiked on VLP vaccine is capable of inducing recall antibodies raised against conserved epitope of HA_2_ during early infection and this conception might form the basis for design of universal vaccine.

In this study, we also investigated the VLP vaccines induced cellular responses and determine whether the cytotoxic T cell lymphocyte (CTL) response is involved in broader protection effect against multiple influenza viruses. Using the ELISpot assays, we compared the Th-1 and Th-2 responses to immunization with VLP vaccines. The data presented in Figure 5A and B demonstrate that all VLP vaccines are capable of inducing the strong Th-2 responses in mice upon stimulation with whole viral antigens of either homologous H5N1 or heterologous PR8 strain, whereas only the H5M2eN1-VLP vaccine can induced detectable level of IFN-γ response when heterologous viral stimulation or infection. Even though the H5M2eN1-VLP vaccine cannot induce significant CTL activity for direct virus-specific clearance, but the protective role of IFN-γ in inhibition of virus replication during the initial stages of infection and contribution to recall heterologous response against influenza virus has been reported [31], [32]. This differentiated cellular response may at least partially explain the superior immunogenicity of the H5M2eN1-VLP vaccine compared to other VLP vaccines, thereby conferring better catching-up immunity once infected with a heterologous viral strain and cause faster recovery from illness.

For development of a better cross-protective influenza vaccine, a recombinant VLP vaccine-based strategy has several notable advantages. Vaccination with VLPs presenting the HA and NA in native conformations similar to the parental virus that stimulated the antibodies will preferentially bind to the oligomeric HA_1_ in humans, which can effectively reduce viral replication in microneutralization assays [33]. Secondly, a cocktail of neutralizing and protective antibodies elicited by H5M2eN1-VLP vaccination would broaden the protective spectrum against a wide-range of influenza viruses. Thirdly, in mice vaccination experiments, reasonable doses of H5M2eN1-VLP vaccine (15 µg) without adjuvant have reduced the fatality against lethal challenge with heterologous and evolutionarily distant strains of influenza viruses (10× MLD_50_), which illustrates that the VLP vaccine presented here is a promising candidate for the future development of a universal vaccine. Finally, the flexibility of a VLP platform permits improved manipulation and design of a better universal vaccine against most of the circulating and emerging influenza virus strains.

## Materials and Methods

### Cells, Viruses, and VLP Vaccine

MDCK cells were obtained from the Bioresource Collection and Research Center, (Hsinchu, Taiwan) and maintained in 1× DMEM (HyClone, South Logan, UT) supplemented with 10% fetal bovine serum (Gibco, San Diego, CA) in a humidified incubator at 37°C with 5% CO_2_. Influenza A viruses A/PR/8/34 (H1N1), A/California/07/09 (H1N1), and reassortant RG-14 (H5N1; HA and NA were derived from A/Vietnam/1203/04, and the remaining backbone genes were derived from A/PR/8/34 virus) were propagated in MDCK or chicken eggs for microneutralization and challenge studies, respectively. Additionally, other H1N1- and H3N2-subtyped viruses used in NA inhibition assay were kindly provided by the collaborator from CDC Taiwan and propagated in MDCK cells. Three types of VLPs were produced in Vero cells and purified as described previously [13]. Equal amount of various VLPs were separated in a 7.5–12.5% gradient gel, and subjected to either Coomassie blue staining or western blot analysis with respective specific antibodies. The relative abundance of HA and NA attributed to total VLP proteins was quantified by FluorChem® HD2 (Alpha Innotech) and Alpha VIEW AS software (Figure S1A, B) and summarized in Table 1.

### Hemagglutinin Inhibition Assay

To assess hemagglutination inhibition (HAI) titers, RDE-treated sera were tested in 2-fold dilutions starting with an initial dilution of 1∶20. Diluted sera were mixed with 4 HA units of corresponding viruses and incubated at room temperature for 40 min, then mixed again with a 0.75% suspension of guinea pig red blood cells. After 2 h further incubation, hemagglutination was assessed by eye. HAI titer is expressed as the reciprocal of the highest dilution that showed 50% inhibition of hemagglutination. A titer of 10 was assigned if no inhibition was observed at a serum dilution of 1∶20.

### Neuraminidase Inhibition Titer (NAI Titer) and Assay

The NAI titer was determined according to the protocol developed by minimization and optimization of the conventional assay [14]. NAI titers were defined as the inverse of the highest serum dilution at which the mean absorbance was ≤50% of the mean signal of virus controls. NAI titers between control and vaccination sera >1.3 fold was considered to be significant [14]. The NA activity inhibition assay was performed using the NA-Star® Influenza Neuraminidase Inhibitor Resistance Detection Kit (Applied Biosystems, Foster City, CA) to measure the residual NA activity of a defined target virus in serum. All reagents were prepared according to the manufacturer’s instructions. Briefly, serum from vaccinated mice was diluted in NA-Star assay buffer, mixed with virus for 20 min at 37°C, and then incubated with 10 µL of NA-Star substrate for 30 min at room temperature. The samples were then analyzed using a luminometer (PerkinElmer Life Science VICTOR^3^, Waltham, MA) after injection of the NA-Star accelerator into each sample with multiple channel injectors. Inhibition of viral NA activity by antiserum was defined as the percentage of NA activity decrease in the vaccinated group compared to the PBS control.

### Microneutralization

*In vitro* determination of virus-neutralizing activities by immune sera were performed with a modified plaque reduction assay under Avicel overlays [34]. Each RDE-pretreated serum diluted from 1∶20 to 1∶800 was co-incubated with equal volume of distinct viruses (about 50–100 pfu/50 µL). The serum/virus mixtures were incubated at 37°C and 5% CO_2_ for 1 h. MDCK cell monolayers (95% confluence), prepared in 96 well plates, were infected with 100 µL/well (in duplicate) of the mixture and viral adsorption was allowed for 1 h in the incubator. After incubation, plates were immediately overlaid with 1.25% Avicel RC591 (A gift kindly provided by FMC BioPolymer), prepared in 1× DMEM with 0.1% BSA and 2 µg/mL TPCK-treated trypsin (Sigma-Aldrich). Plates were incubated at 37°C in a 5% CO_2_ incubator for 24 hr to allow plaque formation. Viral plaques were stained with anti-NP for H5N1 and PR8 (ab66191, Abcam, Cambridge, MA) or anti-HA for CA/07 (11055-RM05, Sino Biological Inc. Beijing, China), and the revealed plaques were scanned and counted by the AID ELISpot Reader System and EliSpot 5.0 iSpot image analyzer (AID, Strassberg, Germany). Microneutralization titers were calculated from the average of duplicate sample wells by extrapolating the inverse dilution of serum that produced a 50% reduction of virus by comparing the total number of plaques revealed in PBS serum and vaccination serum samples.

### Animals, Immunization, and Viral Challenge

Female BALB/c mice (8 weeks old) were purchased from the National Laboratory Animal Center, and randomly assigned to receive different VLP vaccines. VLP vaccines or PBS (as mock control) were given by intramuscular injection into the quadriceps twice 21 days apart. Blood samples were collected 2 week after each immunization. To investigate cognate and heterologous protective immunity, vaccinated mice were challenged intranasally with a lethal dose (10× MLD_50_) of reassortant H5N1 (RG-14), A/California/07/09 (CA/07), and A/PR/8/34 (PR8) influenza viruses at 3 weeks after boost immunization. The mice were monitored daily for 14 days after the challenge for survival and morbidity (i.e. weight loss, inactivity, and body temperature). All animal experiments were evaluated and approved by the Institutional Animal Care and Use Committee of Academia Sinica. Mice were euthanized if they exceeded 30% loss of body weight.

### IL-4 and IFN-γ ELISpot Assays

Spleens were harvested (Day 42) from mock-treated and vaccinated mice and splenocytes were isolated for ELISpot assays (R & D Systems, Minneapolis, MN, USA). Briefly, cells were depleted of erythrocytes by treatment with 1× RBC lysis buffer (BioLegend, San Diego, CA). Following extensive wash with PBS, cells were resuspended in RPMI medium with 10% fetal bovine serum (Gibco, San Diego, CA) and 50 µM 2-mercaptoethanol. Cell viability was determined by trypan blue staining. The cell suspensions (5×10^5^/well) from the spleen were incubated with pre-coated anti-mouse IFN-γ or IL-4 plates and stimulated (24 h) with inactivated virus (10 µg/mL for H5N1 and 4 µg/mL for PR8). Additional wells of cells were stimulated with Concanavalin A (2.5 µg/mL, 1×10^5^ splenocytes/well) as a positive control for IFN-γ ELISpot assay or were mock stimulated as negative control for ELISpot assays. The plates were incubated overnight in a humidified incubator at 37°C with 5% CO_2_. After four times washing with PBS-0.05% Tween, IFN-γ or IL-4 spots were detected by biotinylated IFN-γ or IL-4 detection antibodies followed by addition of streptavidin-alkaline phosphatase and development with BCIP/NBT substrate solution. Spots were scanned and counted by the AID ELISpot Reader System and EliSpot 5.0 iSpot image analyzer (AID, Strassberg, Germany). The results were expressed as the number of spot/5×10^5^ spleen cells.

### Cytotoxicity Assay

Autologous mouse 4T1 cells were used as target cells. The cells were transducted with a recombinant lentivirus expressing NA antigen of PR8 or wild-type lentivirus (empty vector) as a negative control (Figure S2A). Alternatively, the 4T1 cells were infected with PR8 virus at a MOI of 0.01 for 1 day and then used as target cells (Figure S2B). To assess the potential of antigen-specific cytolytic activity of mice splenocytes vaccinated with VLP vaccines, a fluorescence-based cytotoxicity assay was performed with DELFIA europium (Eu) 2,2′:6′,2″-terpyridine-6,6′-dicarboxylic acid (TDA) cytotoxicity assay reagents (PerkinElmer®). In brief, cytotoxic effectors prepared from splenocytes of mice with/without VLP immunizations (Day 42) were *in vitro* stimulated with inactivated PR8 virion for 5 days or *in vivo* challenged with PR8 virus. The previously prepared target cells were washed, labeled with TDA, washed again, and resuspended in RPMI medium containing 10% FBS and 50 µM 2-mercaptoethanol. Target cells (5000 cells) in a volume of 100 µL were plated into each well of 96-well V-bottomed plates, followed by the addition of 100-fold numbers of effector cells in 100 µL of medium. After a 2 h incubation at 37°C, the plates were centrifuged and a 20 µL supernatant from each well was collected and transferred into a flat-bottom plate which contained 200 µL of Eu solution in each well. The Eu forms a stable complex with released TDA in the mixture and generates fluorescence. The fluorescence of EuTDA was measured by a time-resolved fluorometer (PerkinElmer Life Science VICTOR^3^V, Waltham, MA). Percentage of specific cytotoxicity was calculated by the formula: [Experimental release (counts) − Spontaneous release (counts)]/[(Maximum release (counts) – Spontaneous release (counts)]×100. Maximum release was determined by the lysis of TDA-labeled target cells in triplicate wells with DELFIA lysis buffer. Spontaneous release was measured by incubating with target cells in triplicate wells in the absence of effector cells. Results are represented here as the mean ± SEM of triplicate values.

## Supporting Information

Figure S1 **Quantification of HA and NA antigens in VLPs.** The total proteins of purified VLPs and predetermined concentration of purified H5 protein (ab69748, Abcam) or N1 protein (gel purified from N1-VLP) as indicated on the top of each lane were resolved by SDS-PAGE in a 7.5–17.5% gradient gel and subject to western blot analyses by specific antibodies against H5 (A) and N1 (B). The amounts of HA and NA in 0.5 µg VLPs were interpolation calibrated with their cognate standard curves as labeled in Table S1A and S1B. The HA protein contributes 22.5±5.97% and 2.2±0.19% of total proteins in H5N1- and H5M2eN1-VLPs, respectively. The NA protein contributes 10.9±0.28%, 29.5±8.58%, and 38.6±10.91% of total proteins in H5N1-, H5M2eN1- and N1-VLPs, respectively. The HA protein in VLPs or purified H5 protein split into HA_0_, HA_1_, and HA_2_ as indicated. The NA and NA dimmer proteins are labeled on the left.
(PPT)

Figure S2 **Confirmation of antigen expressed target cells used in cytotoxicity assay.** (A) Western blot analysis of 4T1 cells expressed the PR8 NA protein by Lentivirus transduction. The wild-type (vector control) or PR8/NA bearing recombinant lentivirus was used to infect the 4T1 cells at a MOI 2. The next day, the viral supernatant was removed and added the complete growth medium containing with appropriate antibiotics for selection of stable cell line generation. The empty-vector or PR8/NA expressed 4T1 cell were collected and subjected to western blot analysis with specific antibody (N1, Ab 21305, purchased from Abcam). The loaded protein sample was labeled on the top of each lane and the molecular weight of PR8 N1 protein was indicated. (B) Flow cytometry analysis of PR8-infected 4T1 cells. Alternatively, the 4T1 target cells were prepared with infection of PR8 virus (MOI 0.01) for 1 day. The infected cells were stained with collected mouse sera before (pre-) and after PR8 challenge (PR8 post-infection) together with fluorescein isothiocyanate (FITC)-conjugated anti-mouse IgG. After washing, stained cells were analyzed with a BD LSR II Flow Cytometer and collected data were examined using the FACSDiva software.
(PPT)

Table S1 A. The amount and percentage of HA protein in the total protein of VLP. B. The amount and percentage of NA protein in the total VLP proteins.
(DOCX)

Excel S1 **The quantification data for antibodies recalled by PR8 challenge in VLP-vaccinated mice.** Details of band intensity were quantified and analyzed in this spread sheet. *Left*, band intensity of loading control. *Right*, band intensity of HA_1_ and HA_2_. Both experiments were replicated at the bottom of *left* and *right* panels. The vaccine type and pre−/post-challenge that were treated to mice groups are indicated in columns H and U.
(XLS)

Excel S2 **The quantification data for HA_2_ antibody recalled by homologous H5N1 and heterologous CA/07 viral challenges in VLP-vaccinated mice.** Details of band intensity were quantified and analyzed in this spread sheet. *Left*, band intensity of loading control. *Right*, band intensity of HA_2_. Both experiments were replicated at the bottom of *left* and *right* panels. The vaccine type and pre−/post-challenge that were treated to mice groups are indicated in columns H and T.
(XLS)
