# Supplementary material for: A VLP Vaccine Induces Broad-Spectrum Cross-Protective Antibody Immunity against H5N1 and H1N1 Subtypes of Influenza A Virus
Source: PLoS One. 2012 Aug 7;7(8):e42363. doi: 10.1371/journal.pone.0042363 (PMC3413679; doi:10.1371/journal.pone.0042363)
Supplement: Figure S1 — Quantification of HA and NA antigens in VLPs. The total proteins of purified VLPs and predetermined concentration of purified H5 protein (ab69748, Abcam) or N1 protein (gel purified from N1-VLP) as indicated on the top of each lane were resolved by SDS-PAGE in a 7.5–17.5% gradient gel and subject to western blot analyses by specific antibodies against H5 (A) and N1 (B). The amounts of HA and NA in 0.5 µg VLPs were interpolation calibrated with their cognate standard curves as labeled in Table S1A and S1B. The HA protein contributes 22.5±5.97% and 2.2±0.19% of total proteins in H5N1- and H5M2eN1-VLPs, respectively. The NA protein contributes 10.9±0.28%, 29.5±8.58%, and 38.6±10.91% of total proteins in H5N1-, H5M2eN1- and N1-VLPs, respectively. The HA protein in VLPs or purified H5 protein split into HA0, HA1, and HA2 as indicated. The NA and NA dimmer proteins are labeled on the left. (PPT) [file pone.0042363.s001.ppt]

## Slide 1
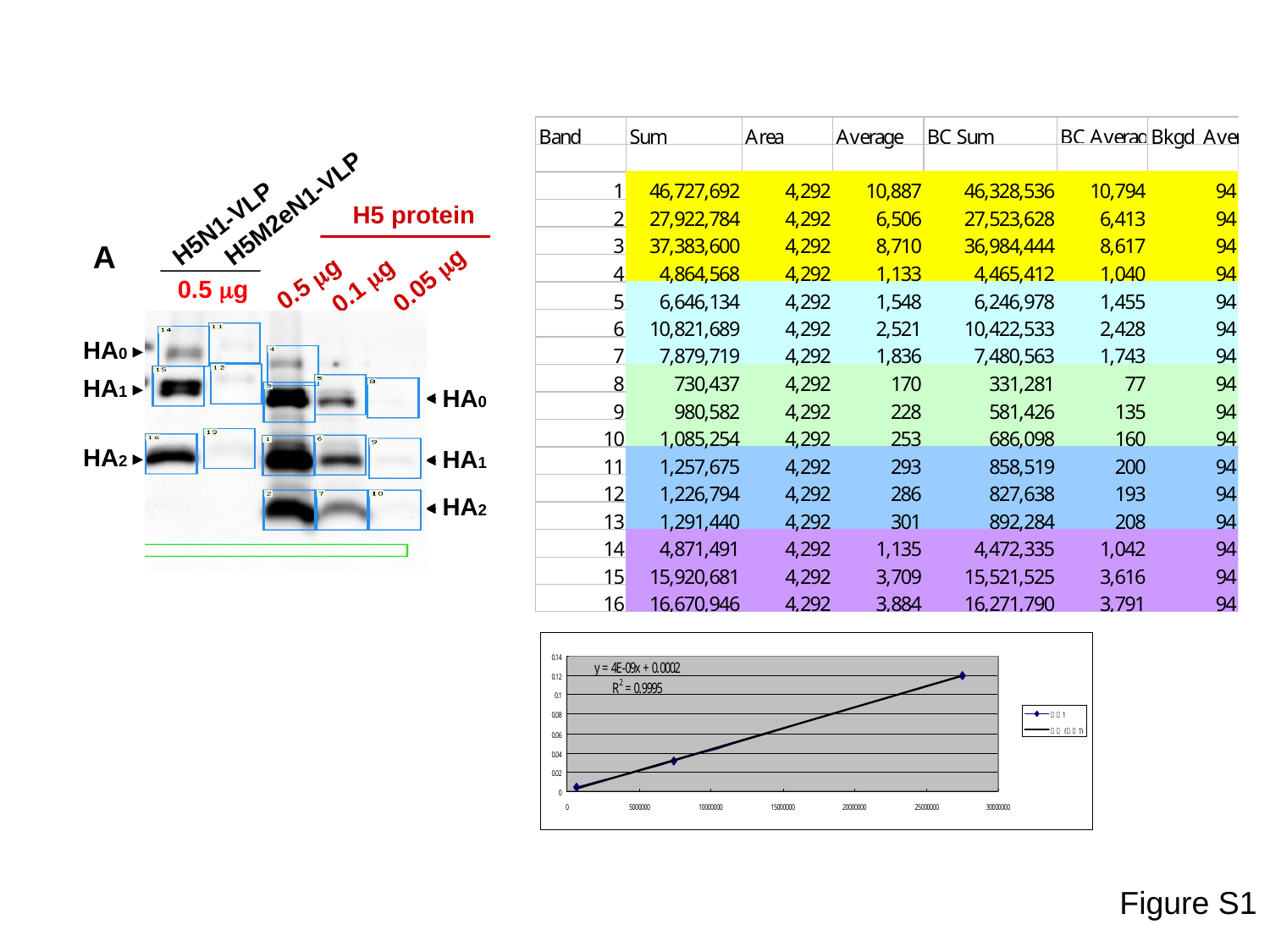

H5M2eN1-VLP
H5 protein
H5N1-VLP
A
0.05 μg
0.5 μg
0.1 μg
0.5 μg
HA0
HA1
HA0
HA2
HA1
HA2
Figure S1

## Slide 2
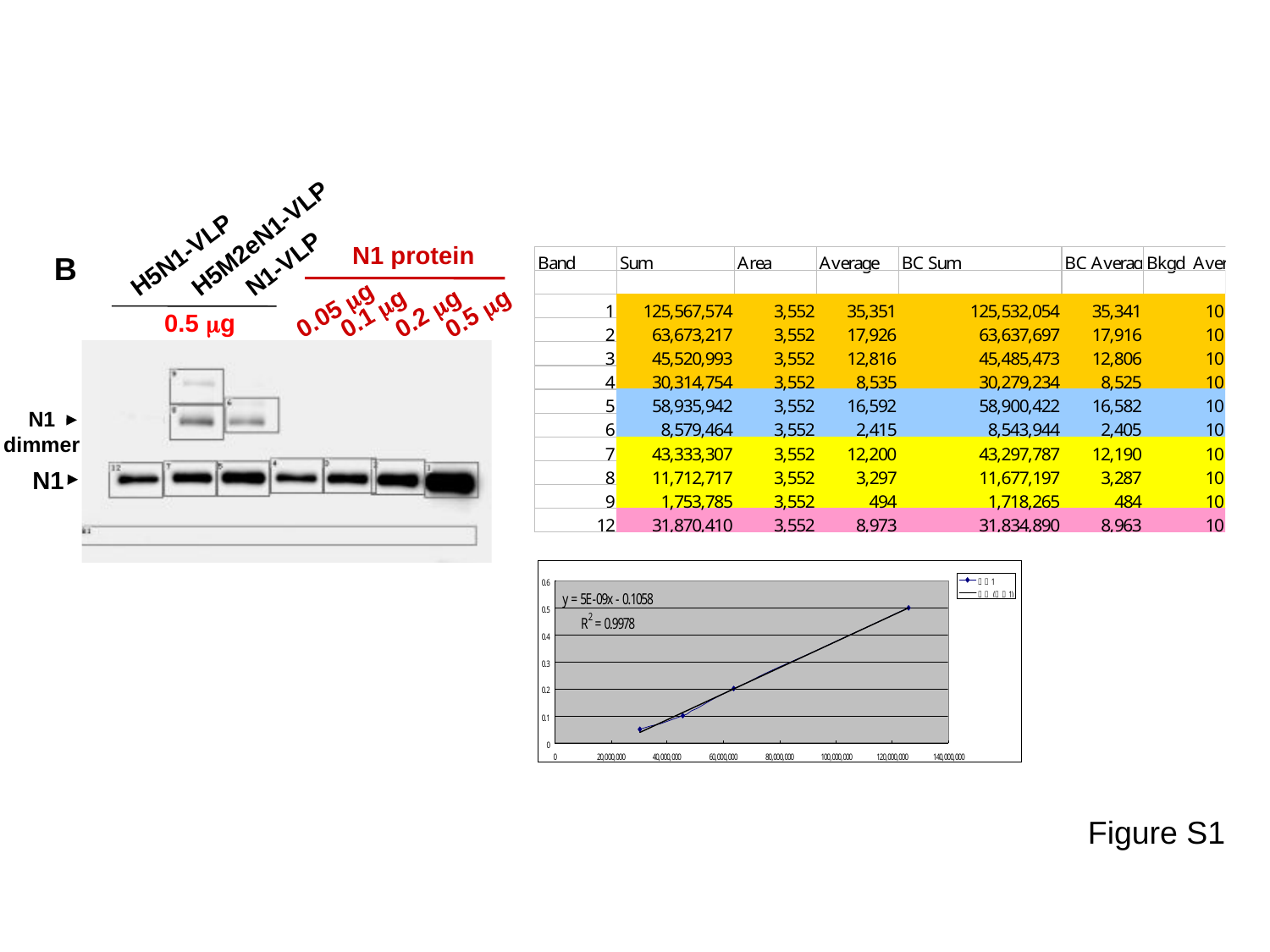

| |
| --- |
H5M2eN1-VLP
H5N1-VLP
N1 protein
N1-VLP
B
0.05 μg
0.1 μg
0.2 μg
0.5 μg
0.5 μg
N1
dimmer
N1
Figure S1
