# Supplementary figures and images for: A VLP Vaccine Induces Broad-Spectrum Cross-Protective Antibody Immunity against H5N1 and H1N1 Subtypes of Influenza A Virus
Source: PLoS One. 2012 Aug 7;7(8):e42363. doi: 10.1371/journal.pone.0042363 (PMC3413679; doi:10.1371/journal.pone.0042363)

## Slide 1
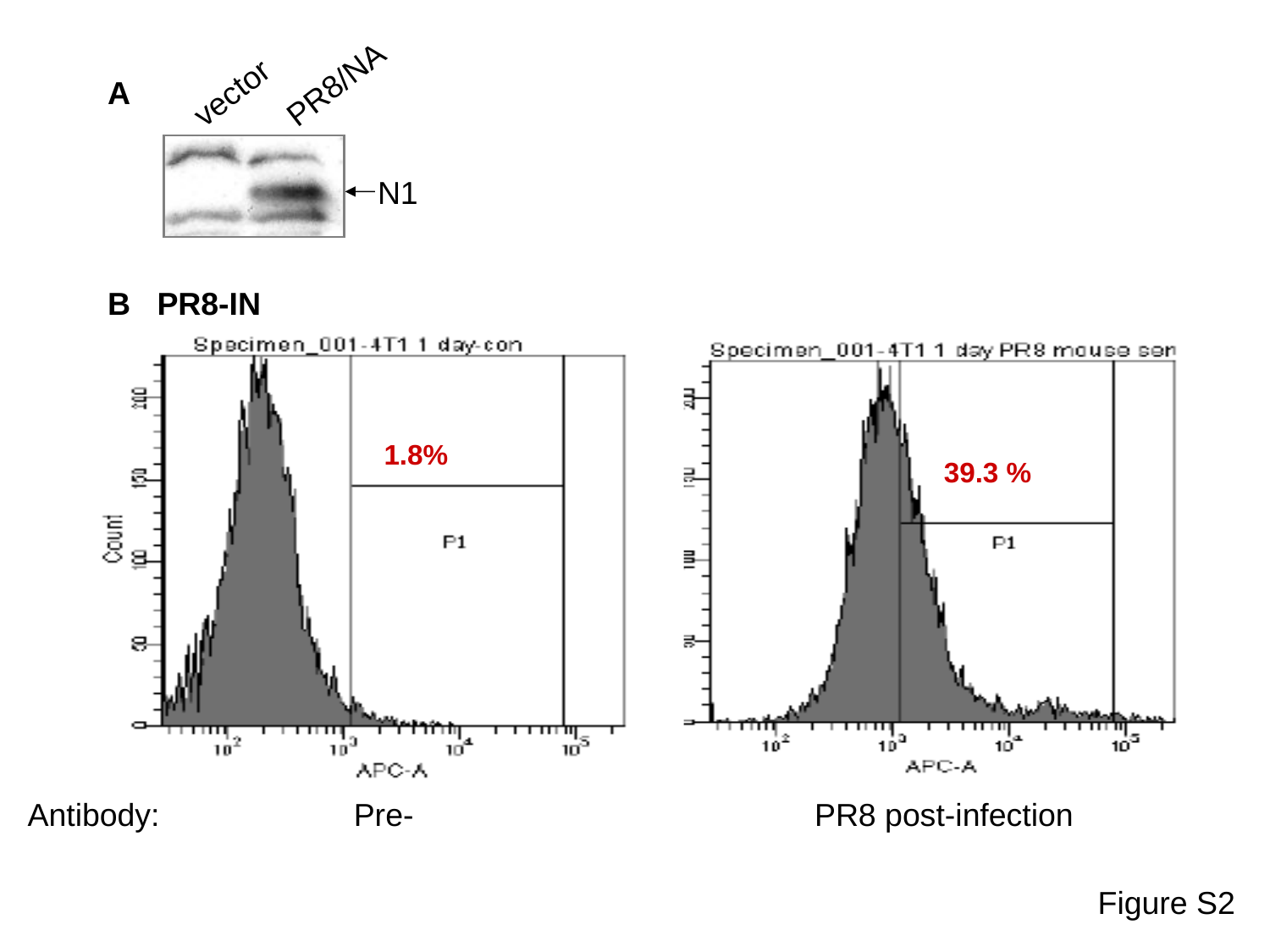

PR8/NA
vector
A
N1
B PR8-IN
1.8%
39.3 %
Antibody:
Pre-
PR8 post-infection
Figure S2

Supplement: Figure S2 — Confirmation of antigen expressed target cells used in cytotoxicity assay. (A) Western blot analysis of 4T1 cells expressed the PR8 NA protein by Lentivirus transduction. The wild-type (vector control) or PR8/NA bearing recombinant lentivirus was used to infect the 4T1 cells at a MOI 2. The next day, the viral supernatant was removed and added the complete growth medium containing with appropriate antibiotics for selection of stable cell line generation. The empty-vector or PR8/NA expressed 4T1 cell were collected and subjected to western blot analysis with specific antibody (N1, Ab 21305, purchased from Abcam). The loaded protein sample was labeled on the top of each lane and the molecular weight of PR8 N1 protein was indicated. (B) Flow cytometry analysis of PR8-infected 4T1 cells. Alternatively, the 4T1 target cells were prepared with infection of PR8 virus (MOI 0.01) for 1 day. The infected cells were stained with collected mouse sera before (pre-) and after PR8 challenge (PR8 post-infection) together with fluorescein isothiocyanate (FITC)-conjugated anti-mouse IgG. After washing, stained cells were analyzed with a BD LSR II Flow Cytometer and collected data were examined using the FACSDiva software. (PPT) [file pone.0042363.s002.ppt]
