# Supplementary material for: A VLP Vaccine Induces Broad-Spectrum Cross-Protective Antibody Immunity against H5N1 and H1N1 Subtypes of Influenza A Virus
Source: PLoS One. 2012 Aug 7;7(8):e42363. doi: 10.1371/journal.pone.0042363 (PMC3413679; doi:10.1371/journal.pone.0042363)
Supplement: Table S1 — A. The amount and percentage of HA protein in the total protein of VLP. B. The amount and percentage of NA protein in the total VLP proteins. (DOCX) [file pone.0042363.s003.docx]

**Table S1A. The amount and percentage of HA protein in the total protein of VLP.**

| **VLP type** | **HA (μg) in**  **0.5 μg VLPs** | **HA (μg) in**  **15 μg VLPs** | **Percentage (%) of HA in total VLP protein** |
| --- | --- | --- | --- |
| **H5N1-VLP** | 0.112 ± 0.030 | 3.37 ± 0.90 | 22.5 ± 5.97 |
| **H5M2eN1-VLP** | 0.011 ± 0.001 | 0.34 ± 0.03 | 2.2 ± 0.19 |

**Table S1B. The amount and percentage of NA protein in the total VLP proteins.**

| **VLP type** | **NA (μg) in**  **0.5 μg VLPs** | **NA (μg) in**  **15 μg VLPs** | **Percentage (%) of NA in total VLP protein** |
| --- | --- | --- | --- |
| **H5N1-VLP** | 0.054 ± 0.001 | 1.63 ± 0.04 | 10.9 ± 0.28 |
| **H5M2eN1-VLP** | 0.147 ± 0.043 | 4.42 ± 1.29 | 29.5 ± 8.58 |
| **N1-VLP** | 0.193 ± 0.055 | 5.78 ± 1.64 | 38.6 ± 10.91 |
